# Immobilized Cells of *Bacillus circulans* ATCC 21783 on Palm Curtain for Fermentation in 5 L Fermentation Tanks

**DOI:** 10.3390/molecules23112888

**Published:** 2018-11-06

**Authors:** Jinpeng Wang, Yao Hu, Chao Qiu, Haoran Fan, Yan Yue, Aiquan Jiao, Xueming Xu, Zhengyu Jin

**Affiliations:** 1 School of Food Science and Technology, Jiangnan University, Wuxi 214122, China; allison_yao@163.com (Y.H.); q930017357@163.com (C.Q.); fanhaoran0622@163.com (H.F.); huyaominoz@gmail.com (Y.Y.); jiaoaq@jiangnan.edu.cn (A.J.); xmxu@jiangnan.edu.cn (X.X.); fpcenter@jiangnan.edu.cn (Z.J.); 2 Synergetic Innovation Center of Food Safety and Nutrition, Jiangnan University, Wuxi 214122, China; 3 State Key Laboratory of Food Science and Technology, Jiangnan University, Wuxi 214122, China

**Keywords:** cell immobilization, palm curtain, CGTase, cyclodextrin, fermentation

## Abstract

Palm curtain was selected as carrier to immobilize *Bacillus circulans* ATCC 21783 to produce β-cyclodextrin (β-CD). The influence for immobilization to CGTase activity was analyzed to determine the operation stability. 83.5% cyclodextrin glycosyltransferases (CGTase) of the 1st cycle could be produced in the 7th cycle for immobilized cells, while only 28.90% CGTase was produced with free cells. When palm curtain immobilized cells were reused at the 2th cycle, enzyme activities were increased from 5003 to 5132 U/mL, which was mainly due to physical adsorption of cells on palm curtain with special concave surface structure. Furthermore, conditions for expanded culture of immobilized cells in a 5 L fermentation tank were optimized through specific rotation speed procedure (from 350 r/min to 450 r/min with step size of 50 r/min) and fixed ventilation capacity (4.5 L/min), relations between biomass, enzyme activity, pH, and oxygen dissolution was investigated, and the fermentation periods under the two conditions were both 4 h shorter. Compared with free cell, immobilized cell was more stable, effective, and had better application potential in industries.

## 1. Introduction

*Bacillus circulans* ATCC 21783 was separated from 600 strains with high activity and alkali resistance (pH 10.0), which produced mainly β-CGTase that created mainly β-CD, few γ-CD, and no α-CD [1,2,3,4,5]. Thus, it offered great advantages for purification of β-CD. In order to have a better operational stability and higher cell concentration during fermentation, cell immobilization is often conducted rather than applying free cells directly [6,7,8]. Immobilization methods could be classified into irreversible immobilizations such as entrapment, covalent binding, and aggregation, and reversible immobilization methods such as ionic binding, adsorption, metal binding, and affinity binding [9,10,11]. Among these methods, physical adsorption is considered as an excellent potential technique due to its nonnegligible advantages (prolonged and repeated use, ease of separation from the fermentation medium, and less contamination caused) [12,13,14].

A large volume of literature has reported the production of CGTase based on cell immobilization. Organic matrices such as loofa sponge, alginate gel, agar gel, chitosan, synthetic adsorption resin [15,16,17,18,19,20,21,22,23], inorganic matrices like SiO_2_/TiO_2_, SiO_2_/MnO_2_ and seas and could also be used as immobilize matrices [16,17,24]. Vassileva et al. [20] used agar gel to immobilize *Bacillus alkaliphilus* ATCC 21783, and the residual enzyme activity of the immobilized cells ranged from 90% to 95% after cultivating for 240 h in a fluidized bed reactor. Large groups of vegetative cells that continued to grow rapidly inside the agar beads were observed through scanning electron microscopy, indicating that high CGTase activity was due to the immobilization of cells. Mazzer et al. [16] produced cyclodextrin by *Bacillus firmus* strain 37 cells. Cells were immobilized by adsorption on silica-titania (SiO_2_/TiO_2_) and silica-manganese dioxide (SiO_2_/MnO_2_) matrices; both of the matrices showed superiority in cyclodextrin yields after incubating in 250 mL Erlenmeyer flasks for 10 days. Production of β-CD was reached to 16.7 mM and 17.3 mM, respectively, in comparison to 8.3 mM with free cells.

The recycling characteristics of cells during the cultivation, which makes the immobilization process environmentally friendly and potentially suitable for industrial scale-up, is of great importance when considering cell immobilization. Pazzetto et al. [25] used loofa sponge, loofa sponge-chitosan, loofa sponge-alginate and synthetic sponge as matrices for the immobilization of *Bacillus firmus* strain 37 cells for cyclodextrin production. Operational stability was evaluated in 4 repetitive cycles for 5 days, all of the immobilized cells showed better operational stability than free cells, the loofa sponge–alginate immobilized cells maintained 77% β-CD production of the 1st cycle in the 3rd cycle, whereas the free cells were almost inactive in the 3rd cycle. Thus loofa sponge–alginate could apply as an environmentally friendly and potentially suitable matrix for β-CD production and industrial scale-up.

Though cell immobilization offers great advantages in avoiding aggregation of free cells, prolonging reuse cycles, improving yields, and resisting inhibitors, the previously reported immobilization carriers display obvious disadvantages, such as dissolution and desorption of cells that could reduce product purity and reused cycles, the growth of aerobic cells could be inhibited due to difficult ventilation after immobilization, structure and diffusion of cells could also be distorted and limited [26]. An inadequate immobilization may even negatively affect the properties of the enzymes [27]. Besides, high industrial cost limits the development of cell immobilization on a large-scale [11,28,29,30]. Thus, selection of matrices is of great importance for cell immobilization due to cost, efficiency and stability issues [31,32].

Recently, fibrous matrices have gained much attention and been successfully used as alternative supports for cell immobilization [23,33,34]. Palm curtain, a plant derivative, is a renewable, readily available, and low-cost material, widely used in scallop feeding, which was considered as a new material for cell immobilization for CGTase. Few literatures have reported the selection of palm curtain for microbial cell immobilization before. In this paper, we investigated the potential of palm curtain as carriers of *Bacillus circulans* ATCC 21783. The optimum immobilization time and fermentation conditions for immobilized cells such as incubating duration, temperature, and pH were determined in flasks in advance. After that, fermentation was conducted in a 5 L tank, the enzyme activity, biomass, pH value, and dissolved oxygen during fermentation were determined and optimization conditions were further explored.

## 2. Results and Discussion

### 2.1. Morphology of Immobilized Cells

**Figure 1:**
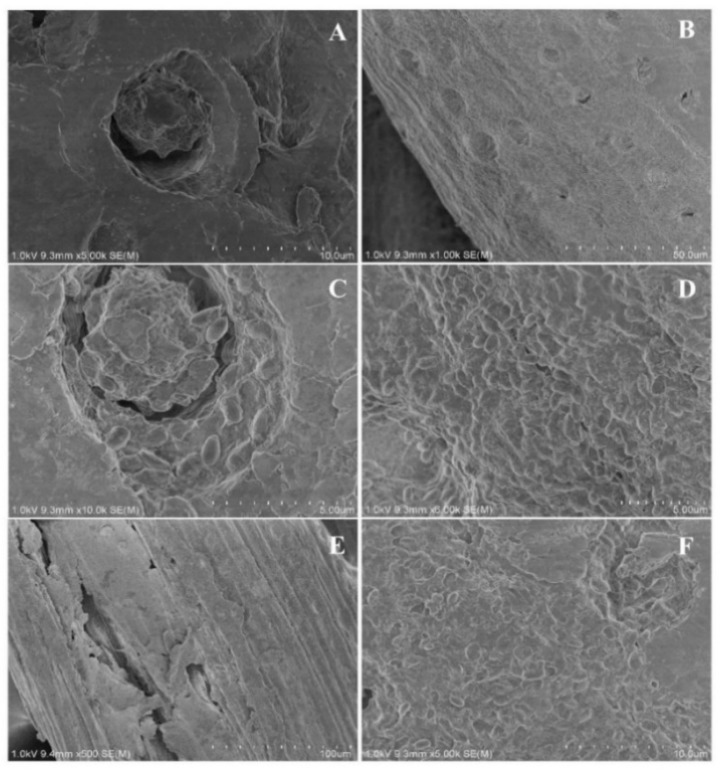
SEM images of different matrices (**Page: 3A**,**B**) initial palm curtain with magnifications of ×5000 and ×1000, respectively; (**Page: 3C**,**D**) *Bacillus circulans* ATCC 21783 immobilized on palm curtain with magnifications of ×10,000 and ×5000, respectively; (**E**) initial loofa sponge with magnification of ×500; (**F**) *Bacillus circulans* ATCC 21783 immobilized on loofa sponge with magnification of ×5000.

Adsorption is an important method for reversible cell immobilization, which is based on the attachment of cells on the surface of carrier via electrostatics, hydrophobic interactions, Van der Waals forces, or hydrogen bonds [11]. The morphology of immobilized cells by palm curtain and loofa sponge are presented in Figure 1. Cavities of different sizes could be observed on the surface of palm curtain, which not only contributed to an increased surface area, but also strengthened the adsorption of cells. Thus, a large number of cells could adsorb on palm curtain with strong interactions. However, no cavities but coarse surface was found on loofa sponge. Though the magnification of loofa sponge was relative lower than palm curtain, more distinctive features were observed. Kourkoutas et al. [35] pointed out that electrostatic interaction was the mainly reason of cell immobilization by adsorption, which is unfixed and hard to measure. Due to larger surface area of palm curtain, the electrostatic interaction between ATCC 21783 and palm curtain was stronger than that between ATCC 21783 and loofa sponge, indicating that palm curtain is a good carrier to immobilize ATCC 21783.

### 2.2. Immobilization Procedure of ATCC 21783

**Figure 2:**
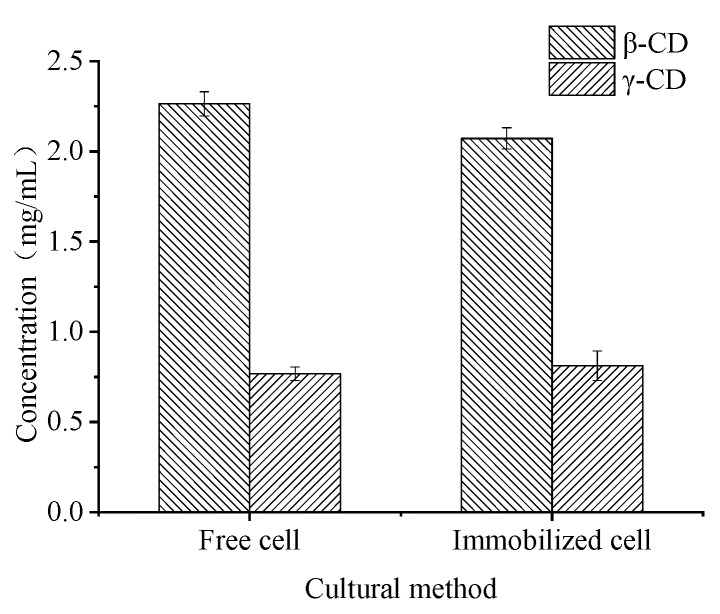
Cyclodextrin production by free and palm curtain immobilized *Bacillus circulans* ATCC 21783.

As the nature of the product of cells should not be influenced by carriers, the products of starch enzymatic hydrolysis by CGTase were determined to investigate the influence of cell immobilization. According to the HPLC detection results, as shown in Figure 2, the products of both immobilized cells and free cells were β-CD and γ-CD, with little difference in the β-CD: γ-CD ratios, which were 73.6:26.4 and 74:26, respectively. The composition and ratios of cyclodextrins were consistent with the results of Vassilevai et al. [4]. Thus, the catalytic properties of CGTase did not change after cells were immobilized on palm curtain.

**Figure 3:**
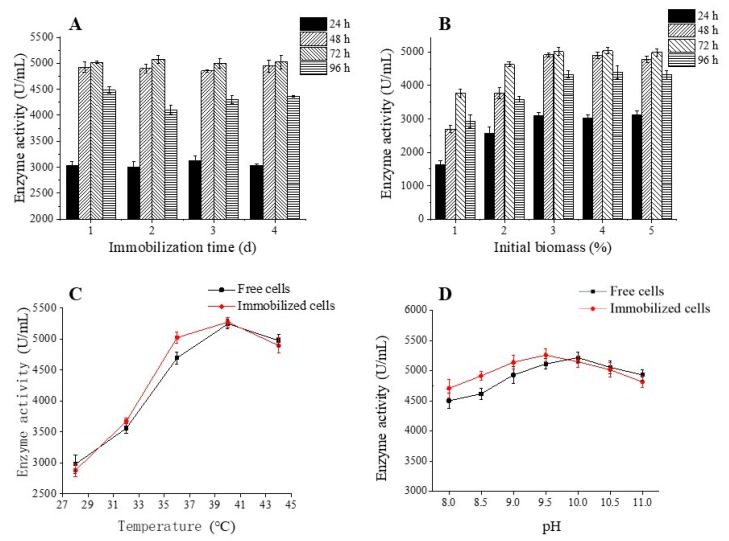
Effect of immobilization time (**A**), initial biomass (**B**), temperature (**C**), and pH (**D**) on the production of palm curtain immobilized *Bacillus circulans* ATCC 21783 enzyme activity.

As physical adsorption was thought to be the main interaction between ATCC 21783 and palm curtain, immobilization time is an important factor that affects the enzyme production. Figure 3A shows the enzyme activities of immobilized cells at different fermentation duration with different immobilization times, where relatively higher enzyme activities were found when the fermentation duration was 48 or 72 h, and no significant difference was observed among cells immobilized for 1, 2, 3, and 4 days while the fermentation duration was conducted for 48 h and 72 h. Thus, the optimum conditions were an immobilization time of 1 d and fermentation duration of 48 h. As can be seen from Figure 3B, when the initial biomass increased from 1% to 3%, the enzyme activity reached the highest value (3%), then remained at the peak level though the initial biomass was further increased to 4% and 5%. This could be attributed to the limited surface area of palm curtain.

The optimum temperatures and pH were also determined for both free and immobilized cells during fermentation. As shown in Figure 3C, the optimum temperatures for both free and immobilized cells were 40 °C, and the enzyme activities were almost same at the optimum temperature, indicating that immobilization on palm curtain did not affect the normal temperature conditions of cells. However, the optimum pH was 10.0 and 9.5 for free and immobilized cells respectively (Figure 3D). A lower optimum pH could attribute to the pretreatment of palm curtain under alkaline conditions, making the palm curtain slightly alkaline. 

### 2.3. Operation Stability of Immobilized Cells

Proper materials to immobilize cells should be safe, nontoxic, mechanically rigid, hard to be degraded by cells, cheap and easy to obtain. Palm is a kind of crude fiber which meets all the properties for being a cell immobilization material. In order to find whether it works or not, palm was cut into palm curtain with size of 2.5 cm × 2 cm × 0.5 cm (length × width × height), and ATCC 21783 was immobilized on it through two ways: physical adsorption and inclusion by sodium alginate.

**Figure 4:**
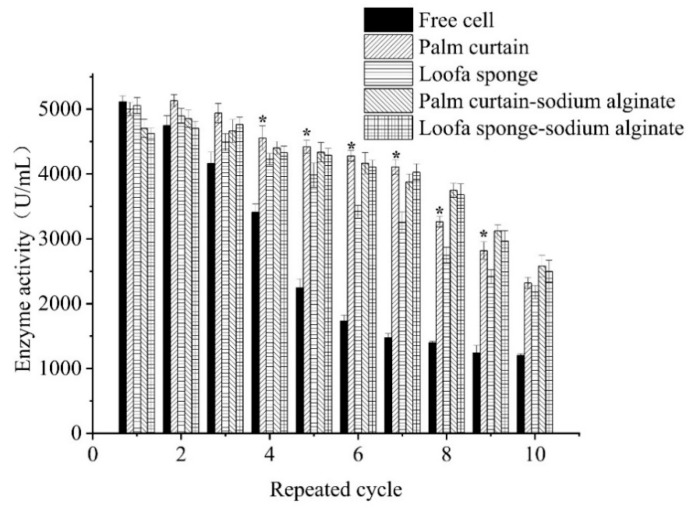
Reusability of different matrices immobilized *Bacillus circulans* ATCC 21783 (* means the significant difference between palm curtain and loofa sponge, *p* < 0.05).

Enzyme activities of free cells and immobilized cells was measured and compared as shown in Figure 4. Significantly higher activities of immobilized cells than free cells were observed when the cells were reused for more than two cycles. More repetitive cycles showed bigger difference and more advantages for immobilized cells. Furthermore, immobilized cells on loofa sponge showed significantly lower activity than that on palm curtain after four cycles, which indicated stronger physical adsorption force of ATCC 21783 on palm curtain than loofa sponge. Pazzetto et al. [25] reported that the strain on loofa sponge was unstable and easily fell off during circular culture because of the weak physical adsorption force.

As can be seen from Figure 4, though higher enzyme activity of free cells was observed at the end of the first cycle than with immobilized cells, palm curtain, palm curtain-sodium alginate, and loofa sponge-sodium alginate immobilized ATCC 21783 showed higher enzyme activities in the following cycles than free cells. In particular, at the end of 2th or 3rd cycle, the enzyme activities of the three immobilized cells were slightly higher than that of the 1st cycle. According to Fernandez-Lopez et al. [36], the mobility of enzymes was reduced due to enzyme crowding, as the chains of enzymes crushed the chains surrounding them, which could improve the stability of enzymes. In this study, cells were crowded and were stable at the initial few cycles, thus resulted in stable production of enzymes. Relative higher enzyme activity in the 2nd or 3rd cycle may result from slightly desorption of cells in fully filled cavities on palm curtain, which led to a larger contact area between cell and fermentation medium, thus more enzymes produced and higher enzyme activity was observed. However, in some cases, immobilized cells usually showed decreased cell activity because of the desorption of cells from carriers [37,38]. 

Among the immobilized cells, palm curtain immobilized cells showed the highest enzyme activity during the 1st to 7th cycle (>80% enzyme activity maintained in the 7th cycle). This tendency could be explained by the relatively stronger adsorption of cells on the surface of palm curtain due to the cavities discussed before. A slow downward trend in the enzyme activities of immobilized cells was observed after the 2th reaction cycle, except for loofa sponge-sodium alginate immobilized cells, the downward trend of which began at the 3th cycle. This downward trend was due to the desorption of cells from the palm curtain and thus enzyme activity in the medium was reduced directly. As a physical adsorption method, desorption is an unavoidable but improvable property in cell immobilization [39]. On the other hand, inactivation of immobilized cells could also result in reduced enzyme activities. Inactivation of cells starts by conformational changes, which may happen in the weakest point of conformation for cells, different conformational changes occur under different conditions [40]. As for palm curtain immobilized cells, pH, oxygen dissolution, and aggregation were nonnegligible factors that may cause conformational changes, inactivation of cells could happen thereafter.

Compared to continuous and rapid declination of enzyme activity found in free cells, inclusion immobilized cells provided more stability as publication showed that ATCC 21783 immobilized on agar maintained 96–97% of the activity after culturing for 300 h [16]. In this study, cells immobilized on loofa sponge-sodium alginate and palm curtain-sodium alginate showed longer circular culture times. However, the immobilization procedure is too complicated to industrialize and no significant superiority in enzyme activities were observed compared to palm curtain. Thus, only palm curtain has potential to be used as an immobilization carrier based on physical adsorption. Several parameters were subsequently determined based on palm curtain immobilized cells.

### 2.4. Biomass and Enzyme Activity during the Growth of Immobilized Cells

**Figure 5:**
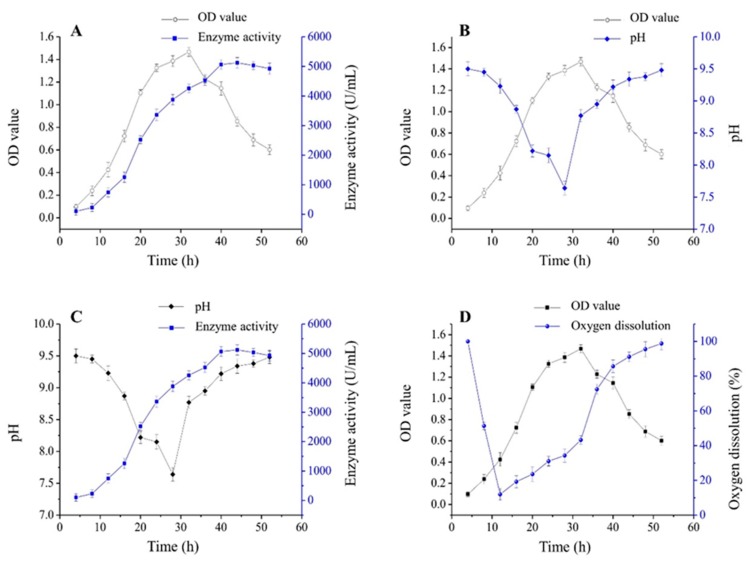
Biomass, enzyme activity, pH, and oxygen dissolution during cultivation of palm curtain immobilized Bacillus circulans ATCC 21783 in a 5 L tank (**A**) relation of biomass and enzyme activity; (**B**) relation of biomass and pH; (**C**) relation of enzyme activity and pH; (**D**) relation of biomass and oxygen dissolution.

Higher enzyme activity should be present at higher cell density according to the theory that more CGTase is produced by more cells [7]. In order to demonstrate the relation between CGTase activity and cell concentration, OD values and enzyme activity were determined every 4 h. As can be seen from Figure 5A, a logarithmic growth phase was observed during 12–24 h for palm curtain immobilized cells, followed by a stable growth phase at 24–32 h and the decline phase began at 32 h. On the other hand, the enzyme activity reached its highest at 32 h. From the results above, it was clear that the cell concentration did not have any significant influence on CGTase production. Similar results were observed by Vassileva [41].

### 2.5. pH Variation during the Growth of Immobilized Cells

Cell immobilization would result in accumulation of metabolites and cause disadvantages for the fermentation. pH is one of the factors which reflect the growth of cells. As can be seen from Figure 5B, the pH value decreased significantly during the logarithmic growth phase, and the lowest pH was observed when the biomass reached the peak, then the pH rebounded gradually to the initial level as the decline phase proceeded. These results provide information that immobilization of the cell on the palm curtain would not affect their growth and metabolites would not accumulate on the palm curtain. During the whole process, enzyme activity increased steadily and was maintained at the peak value when the pH started to rebound (Figure 5C). Thus, the pH value can be used as an indicator of the production of enzyme. Moreover, the figure also indicated that CGTase produced by *Bacillus circulans* ATCC 21783 has a wide range of suitable pH values from 7.5 to 9.5.

### 2.6. Oxygen Dissolution during the Growth of Immobilized Cells

The variation of dissolution oxygen in the process of cell growth is presented in Figure 5D. The dissolution oxygen was reduced sharply before the logarithmic phase, which indicated that the oxygen was consumed with the growth of cells. As time goes on, the dissolution oxygen increased slowly after 12 h. Probably the rotation of the fermentation medium promoted the dissolution of oxygen.

### 2.7. Optimization of the Fermentation Condition

#### 2.7.1. Optimization of the Rotation Speed

*Bacillus circulans* ATCC 21783, as a kind of aerobic bacterium, needs oxygen during growth. As the rotation speed increased, more oxygen was dissolved in the fermentation medium. However, too high a rotation speed (>450 r/min) would result in too many bubbles which is not beneficial for the fermentation. 

**Figure 6:**
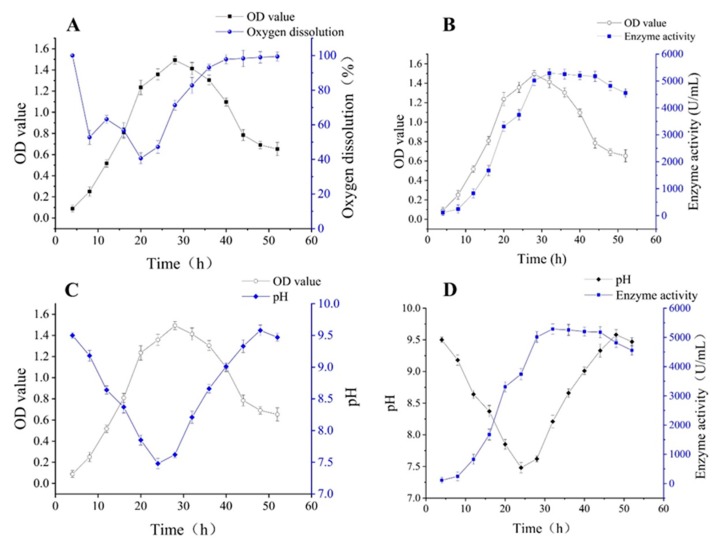
Biomass, oxygen dissolution, enzyme activity and pH variation of palm curtain immobilized *Bacillus circulans* ATCC 21783 by increasing the rotation speed (**A**) relation of biomass and oxygen dissolution; (**B**) relation of biomass and enzyme activity; (**C**) relation of biomass and pH; (**D**) relation of pH and enzyme activity.

As is shown in Figure 6A, the dissolved oxygen was more than 40% under the increased speed procedure conditions. Thereby, the division and propagation of cells were subsequently accelerated. Cell growth entered the logarithmic phase after eight hours and the stationary phases after twenty hours, almost 4 h faster than the original time. The results indicated that the content of dissolved oxygen could be increased appropriately with increasing rotation speed, which is good for the growth of cells. As depicted in Figure 6B, the time cells would take to get to the logarithmic phase and the time to reach the peak value of enzyme production was shortened by increasing the rotation speed, and the stationary phase is still in the peak period for enzyme production.

From Figure 6C,D, the pH value dropped to the lowest at the initial stage of the logarithmic phase, and rebounded to the initial value gradually during the stationary phase. The time for the pH value to achieve the valley was 4 h shorter (from 28 h to 24 h) after increasing the rotation speed. The enzyme activity reached the highest value when the pH value of the fermentation medium was back to the initial level. The results above could be explained by the fact that the dissolved oxygen was enhanced after accelerating the rotation speed, which improved the low state of dissolved oxygen in stationary phases, accelerated the growth of cells, and this process ultimately resulted in less time to reach the valley pH value and peak value of enzyme production.

#### 2.7.2. Optimization of Ventilation Capacity

**Figure 7:**
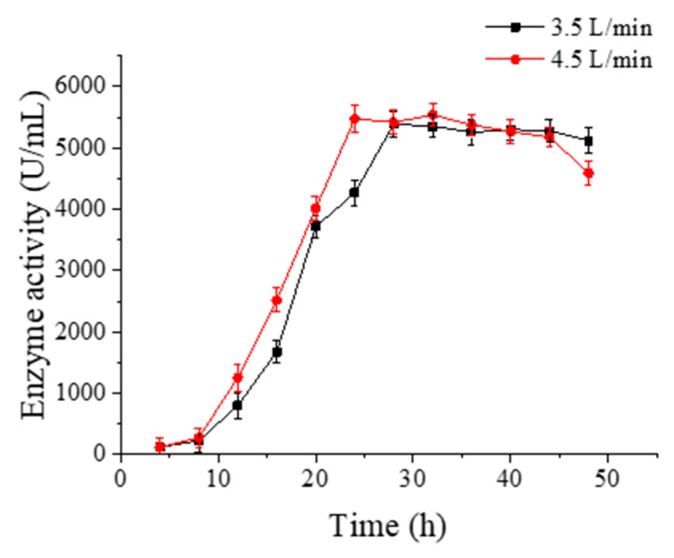
Effects of ventilation on enzyme activity during cultivation of palm curtain immobilized *Bacillus circulans* ATCC 21783 in a 5 L tank.

Different ventilation capacity leads to different contents of dissolved oxygen in the fermentation medium. As the bacterial strain ATCC 21783 is a kind of aerobic bacterium, enough oxygen is needed to promote its metabolism during the proliferation process, especially in the logarithmic phase of growth. Only by increasing the rotation speed could not keep the content of dissolved oxygen at a constant high level. Besides, an excess of bubbles would be produced because of the high rotation speed, making the fermentation medium froth up and overflow from the tank. Therefore, on the basis of the above experiments, the rotation speed was fixed at 400 r/min, the ventilation capacity was set separately at 3.5, 4.5 and 5.5 L/min to improve the microbial growth rate and shorten the enzyme production period f. The effective data when the ventilation was 5.5 L/min are not included in Figure 7 because large amounts of bubbles were produced.

As can be seen from Figure 7, the enzyme activity reached the peak when the ventilation was 4.5 L/min at 24 h, while the enzyme activity took a long time to reach the peak when the ventilation was 3.5 L/min. By comparison, the detected enzyme activity was 3741.83 U/mL and 5539.36 U/mL under 3.5 L/min and 4.5 L/min ventilation, respectively, which demonstrated that sufficient dissolved oxygen was able to shorten the duration of the enzyme production.

## 3. Materials and Methods

### 3.1. Materials

*Bacillus circulans* ATCC 21783 was supplied by the National Bank of Microorganisms and Cell Cultures (Sofia, Bulgaria). All the other chemicals were of analytical grade.

### 3.2. Pretreatment of the Palm Curtain

The pretreatment was performed according to Meleigy and Khalaf [42] with some modifications. The palm curtain was cut into discs about 25 mm (length) × 20 mm (width) × 5 mm (thickness). After soaking in 0.3–0.5% NaOH solution (*w/v*) for 24 h, the discs were immersed in boiling water for 4–6 h and washed thoroughly with distilled water. Then the discs were soaked in distilled water for 24 h, and the water was changed every 8 h. The pretreated palm curtains were then oven-dried at 70 °C and sterilized in an autoclave at 121 °C for 20 min. For comparison, loofa sponge, as immobilization carrier, was cut to the same size and pretreated the same way as palm curtain.

### 3.3. Cell Culture

Cells were grown in culture plates containing solid culture medium composed of (%, *w/v*): 1.0 soluble starch; 0.5 yeast extract; 0.5 peptone; 0.1 K_2_HPO_4_; 0.02 MgSO_4_·7H_2_O; 1.0 Na_2_CO_3_; and 1.5 agar. The plates were incubated at 40 °C for 48 h, and the colonies formed were removed from the culture medium for the preparation of a bacterial suspension, followed by cultivating in seed medium for 24–48 h to expand the fermentation (OD 650 nm 1.0–1.3). The obtained bacterial suspension was freeze-dried and the lyophilized cells were further used for immobilization and as free cells.

### 3.4. Immobilization of ATCC 21783

In the immobilization processes, two pieces of palm curtain or loofa sponge were added to 40 mL culture medium which contained 3% reactivated cells (*w/v*) (OD 650 nm 1.0–1.3) and kept at 40 °C in an orbital shaker at 200 r/min for certain immobilizing time (1, 2, 3, and 4 days) to obtain immobilized cells. Then the immobilized cells were further added into new culture medium and incubated for 96 h, enzyme activities were measured every 24 h. 

As for palm curtain immobilized cells, the optimum immobilization time was selected according to the enzyme activity. Initial biomass (1%, 2%, 3%, 4%, and 5%, *w/v*) and incubation temperatures (28 °C, 32 °C, 36 °C, 40 °C, and 44 °C) were also investigated with an immobilization time of 1 d based on the above procedure. Besides, the influence of initial pH value could have significant effects on enzyme production of ATCC 21783, thus culture media with different pH values (8.0, 8.5, 9.0, 9.5, 10.0, 10.5, and 11.0) were prepared by adding different concentrations of Na_2_CO_3_ solution, a Delta320 pH meter (Mettler-Toledo, Greifensee, Switzerland) was used to measure the pH value precisely. The immobilization processes were same as above (with incubating time of 48 h).

Immobilization of cells in palm curtain or loofa sponge-alginate was carried out according to Phisalaphong et al. [43] with some modifications. Thus 3% sodium alginate solution (*w/v*) was prepared by dissolving sodium alginate in 0.9% NaCl solution (*w/v*) and autoclaved. The cultured cells were collected from the medium by centrifugation, then resuspended in 5 mL of 0.9% sterile NaCl solution (*w/v*), followed by adding the suspension into 50 mL of 3% alginate solution (*w/v*). After that, two discs of palm curtain or loofa sponge were dipped into the alginate-cell mixture, transferred to a 1.47% CaCl_2_ solution (*w/v*) and maintained in gentle agitation for 15 min. The matrices were then rinsed with 0.9% NaCl solution (*w/v*) to get final immobilized cells. 

### 3.5. HPLC Detection of Cyclodextrins

Yield of cyclodextrin was quantified through high-performance liquid chromatography (HPLC). Test conditions are as follows: LC-20A HPLC system (Shimadzu, Kyoto, Japan) equipped with APS-2 hypersil column (4.6 mm × 250 mm, Thermo, Waltham, MA, USA), 70% acetonitrile as the mobile phase, and a refractive index detector (RID) was used to determine cyclodextrin. Detections were conducted with flow rate of 1.0 mL/min, injection volume was 10 µL, and column temperature set as 35 °C. Samples were prepared by centrifugation (10,000 r/min, 15 min, 4 °C) to remove the sediments, followed by filtering through a Millipore filter (0.45 μm). Composition and concentration of cyclodentrins were calculated by comparison with standard samples.

### 3.6. Reusability of Immobilized Cells

After immobilization, carriers were added into 40 mL cultural medium and 10 repetitive cycles of fermentation were conducted. The period for each cycle was 2 days, carriers were washed using 0.9% sterile NaCl solution (*w/v*) after each cycle, after that the carriers were added into a new 40 mL cultural medium for the next cycle. 3% (*w/v*) free cells were cultured in 40 mL cultural medium as blank, cells were removed from the medium through centrifugation after each cycle and added into new culture medium for the next cycle. At the end of each cycle, the enzyme activity was determined.

### 3.7. Cell. Fermentation in 5 L Tank

After being incubated in seed medium containing 3% reactivated cells (*w/v*) in shake flasks for 12 h, fifteen palm curtain discs (10 mm × 7.5 mm × 0.5 mm, length × width × thickness) were transferred to a 5 L fermentation tank, the suspension was then kept at 40 °C with constant stirring (300 r/min) and ventilation (2.5 L/min). Optimization of the fermentation conditions was carried out as follows: the rotation speed was increased when the dissolved oxygen of fermentation medium was less than 50%, from 300 r/min to 450 r/min with step size of 50 r/min; ventilation capacity (3.5 L/min, 4.5 L/min, 5.5 L/min) was detected at a fixed rotation speed (400 r/min). The biomass was measured at 650 nm by an UV/Vis spectrophotometer (Mapada V-1800, Shanghai, China). The enzyme activity, pH value, and dissolved oxygen during fermentation were determined every 4 h.

### 3.8. Enzyme Activity Determination

Cells were removed from the culture medium by centrifugation (3000 r/min, 15 min), and crude enzyme solution was obtained after discarding the sediment. The reaction mixture contained 0.2 mL potato amylose, 0.2 mL of 0.2 M glycine-NaOH-NaCl buffer (pH 8.55), and 10 μL of the crude enzyme solution was incubated at 40 °C for 10 min. Then the reaction was stopped by adding in 0.5 mL of 0.5 M acetic acid, after which 3 mL of 0.005% (*w/v*) iodine was added. The adsorption value was measured at 700 nm using an UV/Vis spectrophotometer at room temperature (Mapada V-1800). For the blank, the sample was substituted by distilled water without enzyme. One unit of enzyme activity (U/mL) was defined as the amount of enzyme that allowed 10% of the adsorption value decreased [41]. The activity of enzyme was calculated using the following equation:

$$\mathrm{One}\;\mathrm{unit}\;\mathrm{of}\;\mathrm{enzyme}\;\mathrm{activity}\;\left(U/\mathrm{mL}\right)=\frac{a-b}{a}\times 1000\times c$$

where *a* is adsorption value of blank; *b* is adsorption value of sample; *c* is dilution ratio of sample. 

### 3.9. Scanning Electron Microscopy (SEM) of Immobilized Cells

5 mM CaCl_2_ containing 2.5% glutaraldehyde was prepared from 50 mM Tris-HCl buffer with pH 8.0. The immobilized cells were soaked in it for 24 h and washed using solutions of 30%, 50%, 70%, 90%, and 100% ethanol, then remained in absolute ethanol for further dehydration and dried naturally. The samples were placed on the surface of a double-faced conductive tape and coated with a gold layer. Scanning electron microscope (SEM, S-4800, Hitachi Instruments Ltd., Tokyo, Japan) with an acceleration voltage of 1 KV was used to observe the morphological character of immobilized cells on palm curtain and loofa sponge.

## 4. Conclusions

Palm curtain was selected as a matrix for ATCC 21783 due to its simple, stable, and eco-friendly properties in the production of CGTase. The optimum immobilization time was 1 d, and the optimum fermentation duration, temperature and pH of immobilized cells were 48 h, 40 °C, and 9.5 respectively. The special concave surface structure was thought to be the reason cells were immobilized stably. More than 80% of the enzyme activity of the 1st cycle was maintained even in the 7th fermentation cycle. Besides, reduction of the mobility of enzymes in the initial few cycles caused by protein chains crushing each other could also lead to improved operational stability. Fermentation parameters of biomass, enzyme activity, pH and oxygen dissolution were measured in the tank. Optimization of fermentation was further investigated, and the fermentation period was shortened 4 h by applying increased agitation speed or increased ventilation. These experimental data should provide useful information for cyclodextrin production through cell immobilization by palm curtain and extend the range of carriers available for cell immobilization.
